# How Psychological Stress Affects Emotional Prosody

**DOI:** 10.1371/journal.pone.0165022

**Published:** 2016-11-01

**Authors:** Silke Paulmann, Desire Furnes, Anne Ming Bøkenes, Philip J. Cozzolino

**Affiliations:** Department of Psychology and Centre for Brain Science, University of Essex, Colchester, United Kingdom; Kings College London, UNITED KINGDOM

## Abstract

We explored how experimentally induced psychological stress affects the production and recognition of vocal emotions. In Study 1a, we demonstrate that sentences spoken by stressed speakers are judged by naïve listeners as sounding more stressed than sentences uttered by non-stressed speakers. In Study 1b, negative emotions produced by stressed speakers are generally less well recognized than the same emotions produced by non-stressed speakers. Multiple mediation analyses suggest this poorer recognition of negative stimuli was due to a mismatch between the variation of volume voiced by speakers and the range of volume expected by listeners. Together, this suggests that the stress level of the speaker affects judgments made by the receiver. In Study 2, we demonstrate that participants who were induced with a feeling of stress before carrying out an emotional prosody recognition task performed worse than non-stressed participants. Overall, findings suggest detrimental effects of induced stress on interpersonal sensitivity.

## Introduction

In his novel *Player One*, Douglas Coupland nicely outlines one of the most challenging social communication issues: “Life is often a question of tone: what you hear inside your head vs. what people end up reading or hearing from your mouth” [1]. Accordingly, a growing body of research has explored how emotions are recognized from speech and how emotional prosody is anchored in the brain (see e.g., [2] for a review). Interestingly, little is known about how psychosocial factors such as depression, hopelessness, or stress can affect the perception and production of vocal emotional attributes. This lack of research is surprising given the prevalence of these factors and their potential to negatively influence social health and well-being. Moreover, it has been shown that stress can affect neural responses to *visual* emotional stimuli [3;4]. Thus, the effects of stress on vocal emotion communication warrant investigation. Hence, the present research set out to explore the effects of laboratory induced stress on emotional prosody from both the sender and receiver perspective using a modified version of the Brunswik [5] lens model introduced by Juslin and Scherer [6] as a theoretical framework. This approach allows systematic exploration of the relationship between acoustic cues used by the sender and perceived by the listener. In this research, ‘stress’ is loosely defined as a state of the organism in which its “internal balance” is disturbed, demanding “an adaptive coping response to restore it” ([7], pg. 172). It cannot be emphasized enough that the ability to accurately de- and encode emotional intentions is crucial in social communication (see, for example, [6]). Misperception of vocally expressed emotions can heavily influence interactions (e.g. frustration experienced by the sender; social isolation experienced by the receiver due to failure in successfully reading vocal emotions). Similarly, vocal misuse, that is improper voice usage (e.g., increased or decreased use of pitch/intensity), during emotional prosody production will likely lead to lower “quality” of emotional speech (this lower quality will result in speech from which emotions are harder to detect).

### Extended lens model of vocal emotions

**Fig 1:**
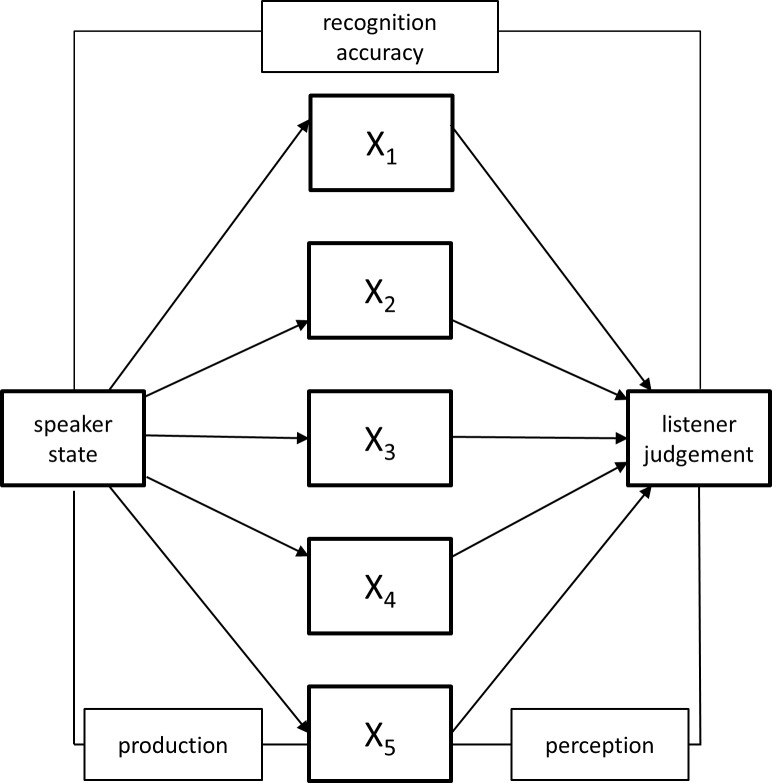
Lens model of vocal emotion expressions. The model describes the relationship between speakers’ production of vocal cues and listeners’ utilization of these cues.

From a theoretical point of view, the speaker/listener interaction can be described in terms of the Brunswik [5] lens model (see Fig 1). Here, we will use an extended version introduced by Juslin and Scherer [6]. Their model aims to describe how speakers express communicative intentions (e.g., emotions) through use of a number of different, inter-correlated acoustic cues. Essentially, cues are considered probabilistic since they cannot be considered foolproof indices of intended expressions. That is, listeners can gauge communicative intentions based on a “flexible” approach when combining the partly redundant available acoustic cues. Hence, the acoustic cues used by speakers do not necessarily *all* map onto judgments made by listeners. Moreover, Juslin and Scherer [6] suggest that “the perceiver infers not simply the emotion as such, but also the speaker’s cognitive appraisal of the actual situation” (p. 85). Obviously, the speaker’s assessment of a given situation will affect their speech production mechanisms. For example, in the case of stress, it can be assumed that physiological indicators linked to stress (such as shortness of breath or muscle tightening) can lead to increased tension in the speech musculature which in turn affects the speech output. Thus, the extended framework characterizes not only the speech production and perception process, but also allows exploring “contextual” effects impacting on speakers’ cue use. These relationships can then be described by means of multiple regression or path analyses (see e.g., [8]) in an attempt to characterize the communication process. Here, we will use multiple regression mediation analyses to describe the link between speakers’ emotional communicative intentions and listeners’ perceptions of those as well as the effect of stress on these processes. Within this framework, we hypothesize that the production of vocal emotional attributes is influenced by speakers’ stress level and that the state of speakers (i.e. their stress feeling) can be identified and recognized by the listener. Given the lack of research to investigate the effect of stress on emotional communication in a combined (sender/receiver) approach, we will summarize research that has explored these effects separately in the following paragraphs.

### Effects of induced stress from the senders’ perspective

In the first part of this investigation, we explore how stress affects emotional prosody production abilities. To our knowledge, this is the first study to explore how the stress level of the sender affects judgments about this speaker by the receiver. This kind of research, however, is crucial given the importance of spoken communication in organizational (e.g., employers with different mental health issues working together), educational (e.g., teachers can be stressed but need to interact successfully with students), health (e.g., communication of medical news from a stressed doctor), and interpersonal (e.g., friendships, marriages) settings. Even though the influence of stress on *emotional* prosody production has not yet been explored, there is evidence in the literature that stress affects speech production abilities in general, i.e., instances where speakers were not asked to produce a basic emotion (see e.g., [9;7;10] for reviews). Interestingly, studies exploring these abilities have failed to describe one single acoustic profile for stressed speakers, but instead describe that primary acoustic parameters (e.g. pitch, loudness) are subject to large variability (increase/decrease) when speakers are stressed (e.g., [11]). Similarly, a recent study that has investigated the effects of stress on speech fluency reports that participants who were stressed paused more frequently during speaking than non-stressed participants. Surprisingly, they also produced more words than non-stressed participants, again suggesting that stress can affect speech production abilities [12]. Some related research on anxiety and its influence on speech production also showed that different acoustic parameters are influenced by feelings of anxiety. In particular, it was shown that increased pitch was linked to increased self-reported anxiety [13]. In a recent review on this topic, Giddens et al. [14] found that physiological changes (e.g., increase in heartbeat, muscle tension) caused by stressors seen to underlie changes of the voice. Thus, if induced stress has an effect on emotional speech production similarly to the impact it has on speech production that does not aim to convey a basic emotion (e.g., [14;15]), differences in the use of acoustic cues (i.e., vocal cue misuse) between speaker groups who differ with regard to their stress level can be expected. In particular, differences in pitch and intensity (or sometimes referred to as loudness) use can be hypothesized based on these previous findings. However, the compelling question that needs to be explored is whether or not listeners are actually influenced by these potential cue use differences. That is, are listeners able to detect that a speaker is stressed when they are expressing emotional speech (Study 1a)? It is also important to investigate if they have difficulties recognizing the emotional tone of voice when speech comes from a stressed speaker (Study 1b). If so, findings would suggest clear detrimental effects of induced stress on producing emotional prosody.

### Effects of induced stress from the receivers’ perspective

A range of studies have investigated how well emotions can be inferred from vocal cues (e.g., [16;17;18;19]) generally, these studies report recognition rates that exceed chance, meaning that listeners are rather accurate at forming impressions about the emotional state of an unknown speaker. For instance, in an emotional prosody recognition study using acted English pseudo-speech, we report accuracy rates for native English speakers of around 75% although rates differed significantly for individual emotions (happiness was least well (48%) and anger was best (91%) recognized; [20]. To date, no study has looked at the effects of induced psycho-social stress on emotional prosody recognition; however, results from individuals with chronic posttraumatic stress disorder (PTSD) revealed significantly impaired emotional prosody comprehension [21]. Whether or not lab induced stress can have a similar detrimental effect will be tested here.

A few studies have looked at emotional *facial* recognition in stressed and non-stressed participants. For instance, Hänggi [22] reports lower decoding of emotional facial expressions in stressed as opposed to non-stressed participants. Similarly, data reported by Herridge and colleagues [23] suggests that men who scored low on the Cook Medley Hostility scale and who were stressed through a cold pressor stressor perform slightly worse in recognizing emotional facial expressions than non-stressed participants. Stress does not only seem to influence facial emotion recognition *accuracy*, but it has also been reported that stressed participants respond more quickly when identifying emotions [24].

The influence of stress on other cognitive functions (e.g., emotional memory) has been explored in more detail revealing mixed findings (e.g., better or worse emotional recall of events, see [25;26; 27;28;29]). Applied stress intensity and task demands will likely play a moderating role in the effects of stress on cognition [30]. According to Sandi [30], enhanced cognitive functions are found when stress intensity is mild and task demands are low, while impaired cognitive functions show when stress intensity and task demands are high.

Study 2 investigates how stress affects emotional prosody recognition abilities. Building on the literature on vocal emotions, an attempt was also made to overcome some previously highlighted ‘limitations’ of investigations studying vocal affect. For instance, while studies using materials portrayed by actors have clear advantages as they test controlled, good quality, stereotypical portrayals, their use has also been questioned (e.g., [9;10]). Additionally, recognition of stimuli spoken by only a small number of actors (usually < = 4) have been tested in the past (e.g., [17;31;18]). This naturally limits generalizability and the kind of inferences about individual emotion recognition patterns that can be made. Here, we therefore decided to use materials spoken by a larger number of *untrained* speakers as this can help determine if previously reported high recognition rates of vocal stimuli are primarily linked to the fact that actors might exaggerate their vocal displays due to their acting training (i.e., they produce highly prototypical voice exemplars).

## Materials

Emotionally intoned speech samples from untrained speakers were recorded to explore how stress affects emotional prosody de- and encoding abilities. The focus of the first two studies was to see whether listeners a) can recognize that a speaker sounds (mildly) stressed when expressing emotions (Study 1a), and b) are worse at recognizing emotions from speech produced by stressed speakers as opposed to non-stressed speakers (Study 1b). The focus of the second study was to explore how listeners’ stress affects recognizing emotional speech samples. To confirm the suitability of created materials, the data were also acoustically analyzed to investigate whether emotional production differences would manifest at the acoustical level. Specifically, we explored how the acoustic profile of an emotional tone of voice changes when speakers are mildly stressed. If stress indeed negatively influences emotional speech production, this should be apparent in both acoustic and perception data (see above). In particular, listeners should be worse at recognizing emotions from speech produced by stressed speakers compared to emotional speech produced by non-stressed speakers. Analyses of acoustics also allowed us to use the extended lens model as a framework to study the influence of stress on emotional prosody. In particular, we ran mediation analyses to investigate how acoustic cues could predict listeners’ performance (see below for further details).

### Speakers

An opportunity sample of eleven (nine females, 19–21 years old) native English undergraduate students was recruited. The goal was to collect recordings from at least ten speakers to obtain a larger number of speakers than usually published for emotion production studies (generally four or less speakers, see e.g., [17]). These participants were randomly allocated to two speaker groups, one group that received a stress manipulation before the emotional prosody production task and one group that received no such manipulation. Given the low number of male volunteers and given that both of them were randomly allocated to the same stress induction condition (see below), both male speakers were excluded from further analysis.

### Stimuli

Fifteen semantically neutral sentences (ranging from four to six words in length) were created (e.g., “The fence was painted brown”; “The bird flew over the house”). We chose to use neutral sentences (rather than pseudo-sentences as frequently used in emotional prosody recognition research) because pilot data indicated that untrained speakers find it difficult to utter pseudo-sentences in an emotional tone of voice without making mistakes. The sentences can be found in S1 Text.

### Recording Procedure

Speakers were asked to read out all sentences in an angry, disgusted, fearful, happy, pleasantly surprised, sad, or neutral tone of voice. For each participant, 105 utterances were recorded (15 semantically neutral sentences x 7 emotions). Specifically, they were asked to imagine a situation in which they had felt the specific emotion before they were presented with the sentences which they had to intone. Recordings were blocked by emotion and the emotion that speakers started with was randomized across speakers. Following previous procedures (e.g., [17]), speakers were not provided with examples of how emotional expressions should sound. Sentences were recorded in a sound attenuating chamber using a high-quality microphone (Blue Snowball) and digitized at 44.1 kHz using the software Audacity (version 2.0.5)

### Stress Induction

To induce feelings of stress in speakers, a sub-part of the frequently used Trier Social Stress Test [32] was administered. Five of the female participants randomly allocated to the ‘stressed’ group were instructed to perform a mental arithmetic task for five minutes. Specifically, they were asked to count backwards from 1022 in steps of 13. Upon giving an incorrect answer, they were informed that their answer was incorrect and required to start again from 1022. The remaining four female speakers allocated to the ‘non-stressed’ group did not have to fulfill this task. A visual analogue scale, ranging from 0–15, was used to measure subjective levels of stress and all speakers indicated stress levels on two occasions: before and after the recording session. Only stress-induced speakers additionally indicated their stress levels right after the mental arithmetic task (see results below). Thus, task order was as follows: after untrained speakers provided informed consent, they were asked to indicate their stress levels for the first time. Next, they received their task instructions and started the recording session. At the end, they provided their stress levels again. Only stress-induced speakers had to perform a mental arithmetic task before the start of the recording procedure and were thus asked to indicate their stress levels at the end of this task, too. All speakers were debriefed at the end of the recording session which lasted approximately 20 minutes.

### Stress Induction Results

Levels of stress for each stress score measurement point were averaged across speakers for each group separately. Descriptive results revealed that speakers allocated to the non-stressed group did not seem to differ between the stress scores obtained at the start of the recording session (4.8) and the end of session (4.1). Initially, participants allocated to the stressed condition also indicated relatively low levels of stress (3.9), but right after the stress induction, their scores increased (9.9) and reduced slightly over time (6.7 as measured at the end of the session). These patterns were statistically analyzed using a series of paired samples t-tests. For the stress-induced group, a paired samples t-test revealed a significant difference between the stress-scores obtained before and after the stress induction, *t*(4) = 3.776, *p* = .02. Speakers felt significantly more stressed after the stress-inducement procedure, indicating that the stress induction paradigm worked as anticipated. No significant differences were found between stress-scores after the stress inducement and the end of the recording session (*p* = .18). Similarly, no significant difference was found between stress scores at the beginning and end of the recording session for non-stressed speakers (*p* = .7). Taken together, these results confirm that the stress induction applied influenced speakers’ subjective stress rating.

### Material Selection

Traditionally, human judges are used to pre-select prototypical exemplars of auditory emotional stimuli before they undergo acoustical analysis or before they are used in a recognition study. However, given that there were no obvious guidelines for how *emotional* sentences spoken by stressed and non-stressed participants should differ, we decided to first rely on a statistical classification approach in selecting materials to avoid possible biases from judges. Thus, a discriminant analysis was conducted to predict emotional category membership based on seven pre-selected standard acoustical parameters (mean, minimum, and maximum pitch, mean, minimum, and maximum intensity, mean duration). These were entered as independent variables while the intended emotional category (anger, disgust, fear, happiness, pleasant surprise, sadness, or neutral) served as the dependent variable in the analyses. Discriminant analyses were carried out separately for materials spoken by stressed and non-stressed speakers. In total 54.3% of all sentences were correctly classified for non-stressed speakers, while 41.1% of recorded materials were classified correctly for stressed speakers.

For the recognition studies, we aimed to present 40 sentences (20 from stressed and 20 from non-stressed speakers) from each emotional category. In addition to having 40 good exemplars, another aim was to avoid repeating sentence contexts too many times within one emotional category. However, this criterion could not be met by selecting materials from the correctly classified utterances only. Thus, the research team selected additional sentences from the pool of incorrectly classified sentences. These selections were based on the quality (researchers’ subjective impression of the emotion) of the recordings. To fulfill all of our criteria, 15 sentences were added to the pool of selected sentences (140) spoken by non-stressed talkers. For stressed speakers, 27 sentences were added to the pool of materials selected through the discriminant analysis. This resulted in a total of 280 selected sentences (20 sentences each from stressed and non-stressed speakers for each emotional category = 40 sentences x 7 emotions) that served as materials in the recognition studies and which were acoustically analyzed to investigate if materials spoken by stressed and non-stressed individuals differ on the acoustic level.

### Acoustical Analysis of Selected Materials

Praat [33] was used for acoustical analysis of standard acoustic parameters. Specifically, we generated a script that automatically extracted mean, maximum, and minimum pitch and intensity values and also measured utterance duration from all speech samples. Pitch floor and ceiling settings were set between 125 and 650Hz. Acoustic parameters were selected based on the observation that pitch, intensity, and duration are the most commonly studied acoustical features in emotional prosody research (e.g., [20;17]). All variables have also been argued to indicate stress [7,9]. The current study did not explore voice quality cues given the lack of previous research of those variables in the context of stress. These data are available in our supporting information file S1 Dataset (Acoustic Data).

### Results of Acoustical Analysis

Primary acoustical measurements (mean and range [maximum-minimum] of fundamental frequency (F0), and intensity (loudness), mean duration) were calculated with mixed linear models with the fixed factors *Emotion* (7 levels [anger, disgust, fear, happiness, neutral, sadness, surprise]; repeated) and *Speaker Group* (2 levels [non-stressed, stressed]; nested in subjects) and the random factor subject using the SAS procedure “proc mixed” in SAS 9.3 (e.g., [34]). For mean pitch, only a significant main effect of *Emotion* was found, F(6,34) = 34.20, *p* < .0001, indicating that pitch use varied between emotions. For example, the highest mean pitch was observed for pleasant surprise sentences (328 Hz), while neutral materials had the lowest mean pitch (207 Hz). For range F0, the main effect of *Emotion* was also significant, F(6,34) = 12.52, *p* < .0001, indicating that there was more varied use of F0 for some emotions than others. Specifically, highest F0 range was found for utterances expressing pleasant surprise, while lowest F0 range was found for materials intended to express sadness. The interaction between *Speaker Group* x *Emotion* was also significant, F(6,34) = 2.63, *p* < .05, suggesting differences in F0 range use between speaker groups. Follow-up independent samples t-tests revealed no group differences between F0 range use for utterances expressing anger, fear or sadness but significant differences between groups when expressing disgust, *t*(38) = 2.96, *p* < .01, pleasant surprise, *t*(38) = 2.70, *p* = .01, and neutral, *t*(38) = -2.35, *p* < .05. The group difference was marginally significant when expressing happiness, *t*(38) = 1.90, p < .07. For the emotional materials, the non-stressed group used a wider range of F0, whereas for neutral utterances the opposite was found (i.e. non-stressed group used a more limited F0 range). For mean intensity (loudness), a significant main effect of *Emotion* was found, F(6,34) = 17.76, *p* < .0001, indicating that different emotions were expressed using different levels of intensity. For instance, surprise was expressed with the highest intensity (56 dB), while sadness, disgust and neutral were all expressed less loudly (all ~50 dB). With respect to the parameter range of intensity, a significant main effect of *Speaker Group*, F(1,7) = 6.16, *p* < .05, and a significant main effect of *Emotion*, F(6,34) = 13.00, *p* < .0001, were found. In addition, the interaction between the two factors was significant, F(6,34) = 3.94, *p* < .01. Follow-up t-tests revealed no significant differences between groups when expressing anger, disgust, happiness, pleasant surprise, or neutral. However, when expressing fear, the non-stressed group used a wider range of intensity, *t*(38) = 3.82, *p* < .001. The same was true when expressing sadness, *t*(38) = 5.90,*p* < .0001. Finally, for duration, only a significant main effect of *emotion* was found, F(6,34) = 10.00, *p* < .0001. Disgust utterances were longest (1.9 seconds), while materials using a neutral tone of voice were shortest (1.3 seconds). Averaged means for each emotional category and each group separately can be found in Table 1 below. Combined, results revealed that materials obtained by untrained speakers contain discernible acoustic features that could be used by listeners to distinguish between different emotional categories. Vocal variability between stressed and non-stressed speakers was also found for some of the emotions expressed confirming the suitability for the use of materials in this project.

**Table 1:** Acoustic Analysis Results.

Acoustic Parameter	Speaker Group	Anger	Disgust	Fear	Happiness	Neutral	Pleasant Surprise	Sadness
mean pitch (Hz)	non-stressed	223.5	221	288.3	249.4	196.9	323.5	213.1
		(10.8)	(12.3)	(10.8)	(10.8)	(12.3)	(12.3)	(12.3)
	stressed	246	221	277.2	257.3	216.3	331.7	229.9
		(12.2)	(9.7)	(9.7)	(9.7)	(9.7)	(10.7)	(10.7)
mean amplitude (dB)	non-stressed	56.8	49.5	53.8	54.7	51	57.1	50.4
		(1.6)	(1.7)	(1.6)	(1.6)	(1.7)	(1.7)	(1.7)
	stressed	54.5	49.3	52.6	52.3	48.8	55.6	48.1
		(1.6)	(1.5)	(1.5)	(1.5)	(1.5)	(1.5)	(1.5)
duration (seconds)	non-stressed	1.4	1.9	1.6	1.4	1.3	1.5	1.7
		(0.1)	(0.1)	(0.1)	(0.1)	(0.1)	(0.1)	(0.1)
	stressed	1.5	1.8	1.5	1.5	1.3	1.4	1.5
		(0.1)	(0.1)	(0.1)	(0.1)	(0.1)	(0.1)	(0.1)
range pitch (Hz)	non-stressed	235.1	338.7	187.9	256.6	109.4	344.6	125.1
		(28.4)	(32.7)	(28.4)	(28.4)	(32.7)	(32.7)	(32.7)
	stressed	260.8	225.7	231.4	181.6	160.1	287.9	107.3
		(32.6)	(25.4)	(25.4)	(25.4)	(25.4)	(28.3)	(28.3)
range intensity (dB)	non-stressed	52.9	43.8	47.3	42.8	41.5	47	43.9
		(1.6)	(1.8)	(1.6)	(1.6)	(1.8)	(1.8)	(1.8)
	stressed	51.4	42.4	38.7	45	40.6	48.1	35.1
		(1.8)	(1.4)	(1.4)	(1.4)	(1.4)	(1.6)	(1.6)

Averaged means (top rows) and standard errors (bottom rows) of acoustical parameters. Note: Hz = Hertz; dB = decibels.

## Methods Studies 1a and 1b

As outlined above, Study 1a aimed to assess whether materials spoken by stressed participants would also be perceived as *sounding* more stressed when compared to materials spoken by non-stressed participants. Study 1b investigated whether listeners find it harder to judge the emotional tone of voice from a speaker who was mildly stressed prior to emotional prosody production as opposed to a speaker who was not stressed. All studies were approved by the Ethics Committee of the Science and Health Faculty of the University of Essex.

### Participants

Thirty-one participants (mean age: 20.5 years, SD: 3.0; 26 females) took part in Study 1a. All participants participated for course credit. For Study 1b, 66 participants (20.5 years; SD: 3.75; 33 females) volunteered. All participants provided informed written consent.

### Procedure

Participants were seated in a quiet testing room approximately 100 cm away from a computer running Superlab 4.5 software. In a computerized task, participants were first presented with a fixation cross in the middle of the screen before one of the 280 (40 sentences from each emotional category: 20 spoken from stressed and 20 spoken from non-stressed participants) pre-selected sentences was played via loudspeakers or headphones. After each presented sentence, participants of Study 1a were asked to indicate whether the speaker sounded stressed or not by clicking with a mouse on one of two response options displayed on screen. Next, they were asked to indicate how stressed the speaker sounded on a scale from 1 (not at all) to 7 (very much). In Study 1b, participants were asked to indicate after each sentence which emotional tone of voice the speaker had used. To this aim, seven response-alternatives (anger, disgust, fear, happiness, neutral, sadness, surprise) were displayed on screen. An inter-stimulus interval of 1500 ms followed the task(s) before the next trial began. Materials were randomly presented in 7 blocks with 40 sentences each. In both studies, participants were presented with 5 practice trials to familiarize them with the procedure.

## Results Study 1a

To explore whether participants can discriminate between stimuli spoken by stressed as opposed to non-stressed speakers, hit (answering “yes, the speaker sounds stressed” when they really were stressed) and false alarm (answering “yes, the speaker sounds stressed” when they were not) rates were calculated for each participant separately. We then calculated a *d’* score (c.f. Signal Detection Theory) for each participant and ran a one-sample t-test to determine whether participants were able to discriminate between stimuli. Results revealed that our population’s *d’* was significantly different from 0 (indicating chance performance), t(30) = .7.562, *p* < .0001, suggesting that participants were indeed able to detect mild stress from the stimuli presented.

Next, responses to the second task (“How stressed does the speaker sound”) were analyzed by calculating mean stress ratings for correctly identified materials spoken by mildly stressed and non-stressed speakers for each participant separately. A paired-samples t-test revealed that materials spoken by stressed speakers were indeed perceived as sounding more stressed (3.40) as opposed to materials spoken by non-stressed speakers (0.24), t(30) = 27.097, *p* < .0001. To confirm that materials spoken by speakers who were induced with stress were generally rated as sounding more stressed than materials spoken by non-stressed speakers, we ran an additional paired-samples t-test on all (i.e. not just correctly identified) trials. Results confirmed that materials spoken by mildly stressed speakers were perceived as sounding more stressed as opposed to materials spoken by non-stressed speakers, t(30) = 5.768, p < .001. An intra-class correlation confirmed inter-rater reliability (α = .92). No data were excluded from the analyses. All data are available in our supporting information file S1 Dataset (Study 1a).

Finally, a discriminant analysis was performed to infer whether the stimuli contained detectable acoustic differences which listeners could rely on to correctly identify the stress level of the speaker. The analysis was performed treating *speaker stress* (stressed, non-stressed) as the dependent variable and mean and range of pitch, mean and range of amplitude, and duration as predictor variables. The value of the single discriminant function was significantly different for stressed and non-stressed speakers (chi-square = 25.68, df = 5, *p* < .001). The correlations between predictor variables and the discriminant function suggested that mean pitch and mean amplitude were the best predictors of speakers’ stress level. Overall, the discriminant function successfully predicted speakers’ stress level for 62.5% of the items (57.1% correct prediction for non-stressed speakers and 67.9% for stressed speakers). Taken together, results from Study 1a suggest that mild stress can be detected from sentences intoned in different emotional prosodies by untrained speakers.

## Results Study 1b

This study aimed to explore whether emotional prosody recognition is more difficult for materials spoken by stressed speakers as opposed to non-stressed speakers. To this aim, we calculated unbiased hit rates (Hu Scores, see [35]) for each emotional category and neutral. These scores were then arcsine-square-root-transformed before they were submitted to a 7 x 2 ANOVA treating *Emotion* (angry, disgust, fear, happiness, pleasant surprise, sadness, and neutral) and *Speaker* (stressed vs. non-stressed) as repeated-measures factors. All available data were entered into the analysis and data are available in our S1 Dataset (Study 1b). Results revealed a main effect of *Emotion*, F(6,390) = 102.89, *p* < .0001, η2 = .613, indicating that some emotions were easier to recognize than others. As can be seen from Table 2 below, angry stimuli were best recognized, followed by surprise, sad, neutral and disgust stimuli. Utterances expressing fear and happiness were least well recognized. In addition, a main effect of *Speaker* was observed, F(1,65) = 58.93, *p* < .0001, η2 = .476, revealing that materials spoken by non-stressed speakers were better recognized than materials spoken by stressed speakers (.65 vs .57). These main effects were informed by a significant interaction between the two factors, F(6,390) = 84.44, *p* < .0001, η2 = .565, suggesting that emotion recognition might depend on the stress level of the speaker. Indeed, pairwise comparisons for each emotional category revealed that materials spoken by non-stressed speakers were generally better recognized for all emotions except happiness and surprise, all *p*s < .0001. For these two positive emotions, better recognition rates were found for materials spoken by stressed speakers as opposed to non-stressed speakers. Mean arcsine-root-transformed Hu score recognition rates split by speaker group and emotion can be found in Table 2.

**Table 2:** Emotion Recognition Rates.

Speaker Group	Anger	Disgust	Fear	Happiness	Neutral	Pleasant Surprise	Sadness
non-stressed	.96 (.02)	.60 (.03)	.62 (.03)	.40 (.02)	.67 (.02)	.60 (.01)	. 71 (.02)
stressed	.72 (.02)	.49 (.02)	.42 (.02)	.50 (.02)	.44 (.02)	.82 (.02)	.62 (.02)
both	.84 (.02)	.55 (.02)	.52 (.02)	.45 (.01)	.55 (.01)	. 71 (.01)	. 66 (.02)

The table lists mean arcsine-root-transformed Hu score rates (and standard errors in brackets) for each emotional category and each speaker group as well as for both groups averaged together.

Taken together, results from Study 1b suggest that participants find it generally more difficult to recognize a negative emotional tone of voice from a speaker who was put under mild stress before the production task but easier to recognize a positive tone of voice from the same speaker group when compared to sentences uttered by non-stressed speakers.

### Mediation Analysis

Overall, results suggest that acoustic cues can index speakers’ stress level which can be accurately perceived by listeners (as shown in Study 1a). Results from Study 1b further suggest that negative emotions expressed by stressed speakers are more difficult to recognize by listeners than the same emotional intentions expressed by non-stressed speakers. To further understand the relationship between the 1) speakers’ stress level and the acoustic cues used, and the 2) listeners’ emotion recognition and acoustic cues, we performed an additional mediation analysis as proposed by [6]. Applying their extended lens model framework, we explored how acoustic cues mediate the relationship between speaker’s stress level and inferences made about their emotional expression abilities. Given the valence effects observed when investigating emotional prosody recognition accuracy, we ran mediation analyses separately for materials expressing positive and negative (and not including neutral) intentions.

**Fig 2:**
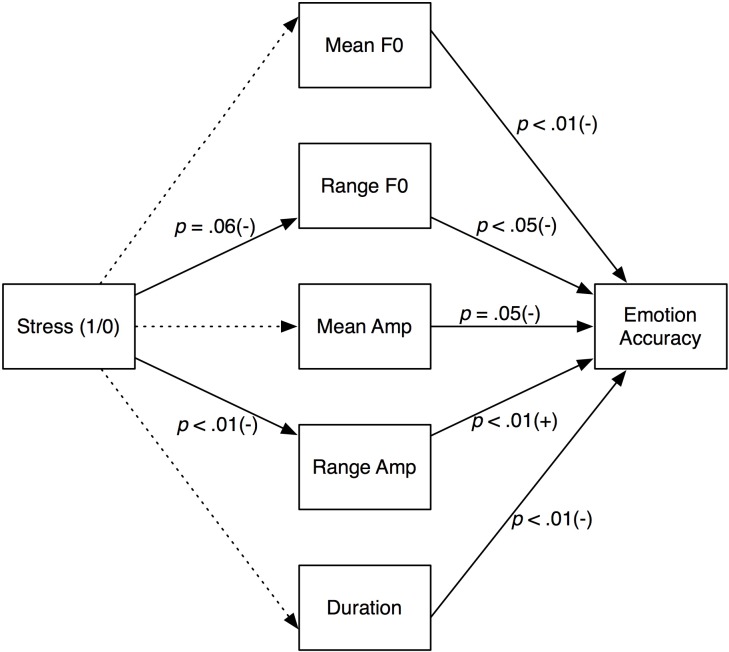
Multiple mediation model for sentences voiced in negative emotions. Dotted paths are non-significant.

To explore the listeners’ accuracy in recognizing the emotions expressed by stressed and non-stressed speakers in the context of Brunswik’s [5] lens model, we utilized a multiple mediation approach that allows, via bootstrapping, for all potential indirect effects to be estimated simultaneously [36]. Focusing first on only those sentences voiced with negative emotions (anger, disgust, fear, & sadness), we specified five key acoustic cues (mean F0, range F0, mean intensity, range intensity, and duration) that could be affected by the stress of the speaker (see Fig 2). Thus, this first mediation analysis simultaneously assessed the effect of stress on our acoustic cues, and whether the indirect effect of those acoustic cues predict accuracy in emotion ratings by the listeners.

As shown in Fig 2, results from 10,000 bias-corrected bootstrapped samples revealed two significant indirect effects, one for range F0 (point estimate = .01, *SE* = .01, 95% bias-corrected confidence interval = [.0003, .04]), and one for range intensity (point estimate = -.05, *SE* = .02, 95% bias-corrected confidence interval = [-.08, -.02]). A contrast test revealed that the indirect effect of range intensity was significantly larger than the indirect effect of range F0 (contrast estimate = .06, SE = .03, 95% bias-corrected confidence interval = [.03, .10]). Stepping through the first indirect path, we see that negative emotions voiced by stressed speakers (coded 1) were expressed with marginally less range in pitch, relative to non-stressed speakers (coded 0), and that less range in pitch was a significant cue for listeners in the accurate detection of those emotions. The second path shows that stressed speakers voiced negative emotions with significantly lower range intensity than did non-stressed speakers, and that lower range intensity as an acoustic cue significantly predicts poorer recognition among listeners of negative emotions. Thus, this analysis seems to inform our earlier finding of listeners finding it difficult to recognize negative emotions voiced by stressed speakers. That is, our second indirect path suggests a ‘disconnect’ between speakers and listeners, in that listeners benefit from greater range intensity in detecting speech containing negative emotions, which is the opposite of how stressed speakers voice negative emotions.

We also conducted a similar multiple mediation model with 10,000 bias-corrected bootstrapped samples on sentences voiced with positive emotions (happiness & pleasant surprise). This analysis revealed no significant indirect effects between the stress level of speakers and emotion recognition in listeners.

## Study 2

This study aimed to investigate if and how experimentally induced stress affects emotional prosody *recognition* abilities. Given the difficulty of recognizing vocal emotional attributes from unknown speakers (i.e. high task demands), we hypothesized to find lower emotional recognition rates for individuals who feel stressed as opposed to those who do not feel stressed when performing the emotional prosody recognition task. Together, data from all studies should provide valuable insights into how stress can influence emotional en- and decoding abilities.

### Participants

An opportunity sample of 55 University of Essex students participated in the experiment for course credit. Data from participants who were not born and raised in England were excluded from the analysis (n = 7), leaving 48 native speakers of English (8 males, age range: 18–21 years). 24 of the 48 participants underwent the stress induction procedure described above.

### Materials

The same materials as used in Studies 1a and 1b were presented.

### Procedure

The procedure was the same as described for Study 1b. Participants who were allocated to the stress condition had to perform the mental arithmetic task (see stress induction procedure for speakers of materials further above) before the start of the recognition experiment. All participants completed the visual analogue stress scale before and after the experiment and participants allocated to the stressed group also indicated their stress levels after the arithmetic task. The overall run-time of the experiment was approximately 40 minutes.

## Results

### Stress Induction

Upon visual inspection of the stress score patterns of participants who underwent the stress induction procedure, it became obvious that a few participants seemed to not be affected by the procedure at all (difference between stress score obtained at the start of the session and after the induction procedure was less or equal to one point on the VAS). These participants were excluded from further analysis given that priming did not work (n = 4). Similarly, some volunteers who did *not* undergo the stress procedure, indicated very high changes between their first and final stress score measurements (larger or equal to five points on the VAS). These individuals (n = 3) were also excluded from further analysis, leaving 20 participants in the stressed and 21 participants in the non-stressed group.

Comparable to Study 1, a series of paired-samples t-tests were then used to investigate whether stressed and non-stressed participants subjectively experienced different levels of stress during the course of the experiment. For the non-stressed group, no difference was found between stress scores obtained at the start of the session (mean 4.71; SD: 3.14) and the end of the experiment (mean: 4.71; SD: 2.72), t(20) = .000, *p* = 1.00. In contrast, participants allocated to the stressed group reported significantly higher levels of stress after the stress induction procedure (mean: 9.5; SD: 3.00) when compared to their scores reported at the start of the session (mean: 2.05; SD: 2.13), t(19) = 8.60, *p* < .0001. The group’s stress levels remained significantly higher throughout the course of the experiment as indicated in differences between stress scores obtained at the start of the session and the end of the experiment (mean: 6.18; SD: 4.37), t(19) = 3.00, *p* < .01.

### Emotional Prosody Recognition Task

This analysis compared emotional prosodic recognition rates between the two listener groups. Accuracy scores were again converted to unbiased hit rates (35). Arcsine-root transformed Hu Scores for the six emotion categories and neutral were then submitted to a 7 x 2 ANOVA treating *Emotion* (angry, disgust, fear, happiness, pleasant surprise, sadness, and neutral) and *Listener Group* (stressed vs. non-stressed) as a between-subject factor. All data were analyzed and can be found in our supporting information file S1 Dataset (Study 2). Results revealed a significant main effect of *Emotion*, F(6,234) = 61.199, *p* < .0001, η2 = .611, revealing higher recognition rates for some emotions than others irrespective of group (see Table 3 showing that anger is recognized best and happiness is recognized worst). A significant main effect of *Listener Group*, F(1,39) = 4.111, *p* < .05, η2 = .095, was also observed showing that listeners who were stressed before the recognition task were less able to recognize the emotional tone of voice of the speakers than listeners who were not stressed prior to the start of the experiment (.65 vs. .59). The two-way interaction between *Emotion* and *Listener Group* was not significant (p = .303).

**Table 3:** Emotion Recognition Rates.

		Response						
Expression	Listener	Anger	Disgust	Fear	Happiness	Neutral	Sadness	Surprise
anger	*non-stressed*	**0.85**	0.16	0.03	0.02	0.07	0.01	0.05
	*stressed*	**0.79**	0.20	0.04	0.02	0.09	0.01	0.04
	*both*	**0.82**						
disgust	*non-stressed*	0.04	**0.56**	0.02	0.08	0.15	0.07	0.17
	*stressed*	0.06	**0.52**	0.04	0.07	0.16	0.06	0.14
	*both*		**0.54**					
fear	*non-stressed*	0.06	0.02	**0.56**	0.06	0.09	0.25	0.08
	*stressed*	0.03	0.05	**0.54**	0.05	0.10	0.24	0.08
	*both*			**0.55**				
happiness	*non-stressed*	0.02	0.01	0.03	**0.46**	0.33	0.02	0.11
	*stressed*	0.02	0.01	0.03	**0.41**	0.35	0.02	0.14
	*both*				**0.43**			
neutral	*non-stressed*	0.01	0.03	0.02	0.02	**0.51**	0.25	0.01
	*stressed*	0.01	0.02	0.02	0.01	**0.53**	0.22	0.01
	*both*					**0.52**		
sadness	*non-stressed*	0.01	0.06	0.05	0.01	0.13	**0.65**	0.01
	*stressed*	0.01	0.05	0.07	0.00	0.14	**0.65**	0.01
	*both*						**0.65**	
surprise	*non-stressed*	0.01	0.02	0.03	0.24	0.03	0.01	**0.71**
	*stressed*	0.01	0.03	0.03	0.23	0.03	0.00	**0.73**
	*both*							**0.72**

This table shows recognition rates (and error patterns) in form of arcsine-root-transformed Hu Scores. The table shows responses given by non-stressed and stressed listeners (and both groups averaged together).

### Error analysis

Table 3 displays errors made by both listener groups when judging emotions from prosody. Visual inspection of the data suggests that both listener groups make similar errors (i.e. no differences in misclassifications). To investigate whether emotional prosody recognition errors could be predicted by acoustic cues, and, crucially whether the overall listener group effect was due to using acoustic cues differently, errors were entered into a discriminant analysis. Sentences were grouped according to their most frequent misclassification; sentences that had equally frequent misclassifications were left out of the analyses (33 for non-stressed, and 37 for stressed listeners). The discriminant analysis for non-stressed participants showed slightly higher prediction accuracy than the analysis for stressed listeners (35.6% vs. 29.6). However, looking at pooled-within correlations between acoustic cues and the different discriminant function scores, no differences were observed between groups. The majority of the variance was accounted for by the first function for both groups (52.6% for the non-stressed listeners, and 47.4% for the stressed listeners) and for both groups, range of intensity had the highest correlation with the first function (.648 and .752). 32.7% (non-stressed) and 37.5% (stressed) of the variance was accounted for by the second function–again, for both groups, the same acoustic variable, namely mean pitch had the highest correlation with this function (.909 and .863). Finally, 11.3% (non-stressed) and 11.7% (stressed listeners) of the variance was accounted for by the third function, and duration correlated most strongly with this function for both groups (.710 and .684). In sum, misclassifications were very similar across different groups and acoustic cue use that led to these misclassifications did not seem to differ between groups.

## Discussion

Interpersonal sensitivity is greatly linked to the ability to accurately de- and encode prosodic cues. The current project set out to investigate the influence of stress on these processes to help illuminate the effects stress can have on emotional communication. Using the extended lens work model as a framework, we first manipulated speakers’ stress levels before expressing vocal emotions. We explored how this manipulation affected acoustic cue use (i.e., we looked at the sender’s perspective). Next, we verified that materials spoken by stressed and non-stressed speakers did indeed contain discernable acoustic cues, and that listeners actually recognized that speakers were either under stress or not. Results from Studies 1a suggest that stress can have an effect on the production of emotional communicative intentions. First, we observed different acoustic cue use between stressed and non-stressed speakers. Second, we saw that naïve listeners can detect whether a speaker was (or was not) stressed while voicing sentences. Using these validated materials, we then explored how the differential cue use impacts on emotion inferences made about the speaker (i.e., we looked at the speaker’s perspective). Results from Study 1b showed that materials spoken by speakers who were stressed before intoning negative emotional sentences were less well recognized than materials by speakers who were not stressed, whereas we observed the opposite for materials spoken in a positive tone of voice. By means of multiple mediation, we further described how acoustic cues mediate the relationship between the sender (stress level) and the listener (emotion inferences made). Finally, looking at how stress affects decoding, rather than encoding, abilities, we showed that recognition of emotional prosodic features is hampered in stressed listeners and that this is unlikely to be attributed to acoustic cues being used differently by the two groups. Overall, these results demonstrate for the first time that stress can affect interpersonal sensitivity. In the following, we will discuss results and integrate them to the wider field of emotional prosody.

### Acoustic analysis

In line with previous studies using acted speech samples (e.g., [16]), findings from our acoustical analyses of materials revealed that different emotions are characterized by different acoustic profiles demonstrating that untrained speakers can convey vocal emotions similarly to trained speakers. For instance, when compared to neutral utterances, actors usually express angry, happy, or fearful utterances using a higher pitch and a louder voice. Here, we observed the same pattern in untrained speakers. Similarly, when compared to other emotions, actors’ sad expressions are often spoken using a lower pitch and less volume, and more slowly than other emotional sentences (for typical actors’ portrayal features see [10]). Table 1 shows that our untrained speakers seem to express sadness in a similar way. Crucially, we report evidence indicating differences between stressed and non-stressed speakers in their cue use when intoning emotional sentences. Specifically, a reduced pitch range was observed for stressed speakers when expressing disgust, pleasant surprise, or happiness. The opposite was true when they intoned sentences neutrally. Additionally, a decrease in intensity range was seen for stressed speakers when expressing fear or sadness. Most previous studies (see [9], or [14] for recent reviews) have explored the effects of stress on ‘non-emotional’ speech (i.e., instances in which the speaker did not aim to convey a basic emotion) production either in the context of deception (i.e., lying is argued to cause stress), or in real-life stressful situations (e.g., air traffic communication). Although these studies have not revealed a uniform acoustical profile for stressed speakers (possibly due to differences in stress and situational contexts, (c.f. [9;7]), many of them report that stressed speakers tend to increase their pitch and possibly their pitch range (e.g., [14]), or intensity (e.g., [37]).

There are, however, studies that do not report an increase in pitch for stressed speakers (e.g., [15;38]). For instance, an investigation on the influence of stress on female voice characteristics showed that women used a lower pitch, lower intensity, and less aerodynamic capacity when speaking in a stressed, challenging situation [15]. These results are comparable to the current data. Here, speakers were also all female and displayed smaller pitch and intensity *range* use for some emotions, suggesting that they possibly displayed lower vocal quality characteristics than non-stressed participants. This argument is supported by the observation that materials were slightly harder to classify for stressed speakers as indicated by the discriminant analysis and lower recognition results in Study 1b for materials spoken by stressed speakers. The variability in pitch and intensity use reported for stressed speakers is argued to stem from their inability to properly control their vocal apparatus resulting in higher variability of acoustic cue use [9]. Emotional prosody production requires speakers to adequately calibrate and adjust their voice depending on the context. Here, mildly stressed speakers seemed to struggle with fine-tuning their pitch and intensity range for some emotional expressions.

Combined, these data from untrained speakers suggest that stress can indeed have an influence on emotional prosody production, resulting in vocal misuse. The data further imply that stress influences production of emotions and neutral utterances. While informative, one limitation of our findings is that it is unclear how results from laboratory generated materials generalize to spontaneous emotional expressions. It might be that spontaneously produced emotional expressions are less (or perhaps even more) susceptible to stress. Thus, future studies should test if findings can be replicated a) even when testing female and male speakers and b) crucially when using different elicitation strategies (e.g., emotion induction procedures before vocal production). Arguably, intoning fearful or sad sentences after a negative emotion induction could lead to larger stress effects than using neutral prosody after a positive emotion induction. In fact, some of the discrepancies reported in the literature (e.g., increase/decrease of pitch and intensity for stressed speech) have been argued to stem from differences in elicitation contexts. For instance, danger or threat related contexts (e.g., pilot conversation, blackouts) seem to result in increased pitch and intensity use, whereas challenging, but not life-threatening situations such as public speaking can result in decreased pitch and intensity use (c.f. [15]).

### Recognizing stress from the emotional voice

In Study 1a, we explored how well listeners are able to reliably detect stress from voices that express specific emotions. As mentioned above, studies investigating acoustic cue use in stressed participants fail to report one single acoustic profile that describes a stressed speaker, though stressed speech generally seems to be distinguishable from non-stressed speech at the acoustic level [7]. The variability in cue use is likely due to differences in stress states of speakers (e.g., high, moderate, low), settings in which speech samples were obtained (real-life, lab), or stress ‘reasons’ (e.g., stress induction differences, threat-related stress, anger-related stress; c.f. [7]). However, irrespective of how or why a speaker might experience stress (and how that manifests at the acoustic level), the question of interest is whether listeners pay enough attention to the potentially subtle changes in acoustic cues to identify that a speaker is under stress. It is important to investigate listeners’ ability to recognize stress from speech samples, as a failure to do so adequately is likely going to lead to misunderstandings and miscommunications. Results from Study 1a suggest that listeners cannot only detect mild stress from unfamiliar voices, but that they can do so even when speakers are expressing specific positive or negative emotions. Thus, the current data support the notion that the acoustic cues used by stressed speakers can indicate that they are experiencing stress while uttering a sentence. Moreover, they also can be taken to support the view that it is not necessarily more difficult for listeners to detect stress in voices that are expressing positive or negative intentions as opposed to relatively neutral intentions. Stress ratings for the speech samples used here further indicate that listeners were aware of the fact that speakers were only “mildly” stressed (i.e., not in a situation of extreme danger) when producing stimuli and intra-class correlations showed that participants largely agreed on their assessment. Results from our discriminant analysis further confirmed that the produced materials contained acoustic features on which listeners could rely when making judgements about the stress level of the speaker. Taken together, these findings add to the growing body of evidence demonstrating that the voice carries extensive information about a speaker (e.g., gender, age, sex, mental health, emotional/affective state; see e.g., [39]) and clearly highlights that this information is used by listeners to assess the speaker’s state of mind.

### Recognizing emotions from the stressed voice

Study 1b set out to examine the implications of stress for speakers who are expressing emotional intentions, that is, are listeners affected by the speakers ‘misuse’ of acoustic cues? Results showed lower emotional prosody recognition rates for materials intending to convey negative emotions expressed by stressed speakers. This suggests that listeners struggle to correctly judge the (negative) emotional tone of voice from someone who is stressed. This difficulty might be linked to stress signaling voice cues overlaying cues crucial for signaling and detecting distinct negative emotions. For example, acoustical analysis of materials used in this study showed that stressed speakers did not modulate their loudness levels as much as non-stressed speakers when expressing fear or sadness. However, this loudness modulation might be vital when trying to accurately identify these emotions. This idea is confirmed by our mediation analysis which showed that stressed speakers’ materials displayed a restricted range of pitch and intensity range; however, while a restricted use of pitch range was associated with higher emotion accuracy, the opposite was found for the restricted use of intensity range. Thus, stressed speakers “violated” a cue use pattern that listeners relied on, leading to lower recognition of their emotional expressions. Alternatively, listeners may simply have picked up conflicting cues from speakers. For example, listeners might expect acoustic cues signaling threat-related stress when listening to fearful stimuli and loss-related stress when listening to sad stimuli–however, instead they picked up stress-related cues triggered by the mental arithmetic task, leading to confusion when trying to assess the speakers’ emotional intention. Either way, the results demonstrate that experiencing mild levels of stress induced through a cognitive task, can negatively affect speakers’ abilities to produce negative emotions leading to lower recognition or identification of their speech samples.

Interestingly, listeners found it easier to recognize pleasant surprise and happiness when expressed by stressed-speakers as opposed to non-stressed speakers. While this finding highlights that stress does not always have to have a detrimental effect on emotional communication, it raises the question why positive emotions should be expressed more “clearly” when under stress. One possible explanation might be that speakers tried to hide their stress levels by exaggerating acoustic cues which are associated with positive emotions. The fact that we do not find main group differences for any of the acoustic parameters speaks somewhat against this possibility, although Table 1 shows that the stressed speaker group generally spoke with a higher pitch (even if not significantly different from the non-stressed group). High pitch has been associated with both surprise and happiness (see [16]); perhaps listeners simply associated high pitch levels with positive emotions (though note that response bias was controlled for in our analyses).

An additional interpretation of this finding comes from the stress and coping literature. For instance, it has been suggested that positive emotions help individuals cope with stress (e.g., [40]). More specifically, it has been claimed that feeling positive helps people bond with others. In severe cases of stress, feeling positive about something might also be used as a “time-out” or relief from feeling stressed [41]. Perhaps then, speakers from the stressed group embraced the opportunity to intone sentences in a positive tone of voice to feel less stressed and thereby helped make their portrayals more convincing than stimuli produced by non-stressed speakers.

In sum, results from Study 1b suggest that a speakers’ stress level influences how well their emotional intentions are recognized by listeners. For the most part, being stressed leads to listeners recognizing negative emotional intentions less well; however, the effect was found to be reversed when expressing positive emotions. As this is the first study to explore the influence of speaker stress on emotional prosody, future studies should be conducted to confirm this pattern with the aim to further investigate the underlying cause of this effect.

### Recognizing emotions when stressed

Study 2 is the first to investigate how listeners’ stress levels can affect decoding emotional prosody. Using materials spoken from untrained, non-professional speakers, we report recognition rates well exceeding chance level (which was set at 14% here), similar to previous studies using materials from actors (e.g., [16]). Crucially, stressed listeners performed worse in this task when compared to non-stressed listeners. This suggests that the ability to perceive paralinguistic attributes of speech deteriorates during stressful situations. Visual inspection of the confusion matrix (Table 3) seems to suggest that both listener groups make similar errors. For instance, anger is most frequently mistaken for disgust, fear is confused with sadness, and happiness is frequently mistaken for pleasant surprise. It has repeatedly been argued that confusion between emotions arises because vocal expressions share some of the main acoustic features (e.g., similar intensity and pitch profile). Also, emotions belonging to the same valence category are easily confused; perhaps less confusions would arise if researchers provided participants with detailed emotion category definitions. The present data suggest that stressed listeners make similar but significantly more errors during emotional vocal recognition when compared to non-stressed listeners. It is unlikely that the stress effect observed here stems from the stimuli used as listeners were presented with a wide variety of materials spoken by both stressed and non-stressed speakers. Instead, it seems as if stress simply hampers the ability to appropriately process the complexity of emotional prosody, though the exact nature of this difficulty requires further investigation. It has been suggested that stress initially leads to heightened processing of (threatening) sensory information, while simultaneously diminishing the ability to process stimuli in more depth (see [3]). Explicit emotional prosodic evaluation requires participants to first extract emotionally relevant acoustic cues, which then need to be combined before a more thorough evaluation of the stimulus can take place (c.f. [2]). If participants fail to sufficiently engage with the latter step of emotional prosody processing, this will lead to a reduction in accuracy rates. Time-sensitive measurements, such as measuring event-related brain potentials, can be applied in future studies to explore more thoroughly the nature of stressed listeners’ inability to judge emotional prosody.

The current data provide no strong indication that prosodic recognition accuracy differed between groups for individual emotions. This suggests that stress generally hampers interpreting emotional auditory cues, a result in line with imaging data reporting comparable brain activation patterns in response to negative and positive facial expressions in stressed participants [3] and a study on chronic PSD patients [21] highlighting that lab induced and chronic stress have a comparable negative effect on the ability to recognize the state of others.

In short, results highlight that even mild stress can affect emotional communication. A failure to accurately interpret emotional intentions of others can lead to interpersonal problems: those who fail to ‘read’ emotions properly may be perceived as unconcerned or indifferent which can then lead to social isolation of those displaying this insensitivity. From a practical point of view then, the current data suggests that speakers and listeners should remember that interpretation of non-verbal acoustic signals is affected by their stress level.

## Conclusion

To conclude, the present investigation has looked at the effects that lab-induced stress can have on the ability to produce and perceive emotional prosody. Results from Studies 1a and 1b highlight that listeners decipher whether speakers are under stress when uttering sentences. Moreover, findings outline that stress can influence how well a speakers’ emotions can be recognized by those to whom they are communicating. When speakers express negative intentions, it seems listeners find it more difficult to recognize those feelings, whereas the opposite was found when stressed speakers expressed positive emotions. Finally, findings from Study 2 demonstrate that emotional recognition is hampered in listeners who are put under mild stress. Combined, these data, for the first time, outline how stress affects emotional vocal communication abilities.

## Supporting Information

S1 Dataset S1 Dataset contains three worksheets. Sheet 1 is called Acoustic Data and contains all variables analyzed for materials generated for Studies 1a, b, and 2. Sheet 2 is called Study 1a and contains all relevant data obtained for Study 1a. Sheets 2 (called Study 1b) and 3 (called Study 2) contain data for Studies 1b and 2.
(XLSX)

S1 Text S1 Text contains a list of the fifteen semantically neutral sentences that were intoned in emotional tones of voice by speakers. (DOCX)
