# Supplementary material for: How Psychological Stress Affects Emotional Prosody
Source: PLoS One. 2016 Nov 1;11(11):e0165022. doi: 10.1371/journal.pone.0165022 (PMC5089770; doi:10.1371/journal.pone.0165022)
Supplement: S1 Text — (DOCX) [file pone.0165022.s002.docx]

**Stimuli**

1. The fence was painted brown.
2. The dog had two owners.
3. The book was green.
4. It was a heavy car.
5. The cat has night vision.
6. The water bottle was full.
7. The shop sells many things.
8. The boxes contained many items.
9. The horse was eating an apple.
10. The bird flew over the house.
11. There was food in the fridge.
12. This is a yellow blanket.
13. The top was made of cotton.
14. The woman crossed the street.
15. The man posted a card.
